# Concurrent child undernutrition indicators in Malawi: application of trivariate logistic regression

**DOI:** 10.3389/fnut.2026.1848833

**Published:** 2026-05-29

**Authors:** Tsirizani Mwalimu Kaombe

**Affiliations:** Department of Mathematical Sciences, School of Natural and Applied Sciences, University of Malawi, Zomba, Malawi

**Keywords:** joint child undernutrition, risk factors, stunting, trivariate logistic regression, underweight, wasting

## Abstract

**Background:**

The sub-Saharan Africa region faces a significant burden of child undernutrition. Indicators of child nutrition are often interrelated; however, previous research has typically analyzed them separately.

**Methods:**

This article investigated the risk of joint child stunting, underweight, and wasting in children under 5 years old in Malawi, utilizing trivariate logistic regression. The study used data from the 2024 Malawi Demographic and Health Survey. All computations were carried out using R software version 4.5.3.

**Results:**

The findings revealed that stunting and underweight frequently occurred together in children, sharing similar risk factors. However, the cases of simultaneous stunting and wasting, as well as underweight and wasting, could not be adequately explained by the available factors. The likelihood of experiencing both stunting and underweight was lower in children aged 12 months and above, those from middle- or high-income households, and those living in rural areas. Conversely, the risk of these two conditions was higher in children with higher birth orders.

**Conclusion:**

Analyzing the risk factors associated with concurrent child undernutrition outcomes provides valuable data for developing child health policies aimed at eliminating multiple forms of undernutrition.

## 1 Introduction

Child undernutrition remains a public health concern in the least developed nations, particularly in sub-Saharan Africa and South-East Asia (1, 2). Undernutrition refers to having insufficient nutrients and energy in a person's body, which can cause adverse effects on body composition, function, and health outcomes (3). This is measured using a quantitative standard normal probability distribution score (z-score) of one of the three anthropometric indicators: height-for-age (HAZ) for stunting, weight-for-height (WHZ) for wasting, and weight-for-age (WAZ) for underweight (4–6). A z-score of less than or equal to -2.0 in any of these measures is considered an indicator of undernourishment in a child (7). Poor nutrition in children can result in stunted growth, developmental issues, and even higher rates of child mortality (8, 9).

Risk factors for child undernutrition include the child's sex, age, birth order, birth interval, breastfeeding duration, promptness of breastfeeding initiation, and whether the child has fever symptoms. Other risk factors include the mother's age at childbirth, occupation, education level, the household's access to clean drinking water, maternal height, household wealth index, and the type of toilet facility used by the household (2, 10–12). For instance, male babies and first- or second-born children are more likely to experience stunting, while younger children are more likely to experience wasting. Additionally, drinking water from unimproved sources is associated with stunting in children (2, 13). Research shows that timely and adequate interventions for child undernutrition can prevent negative consequences and promote catch-up growth in later stages of life (9). To devise appropriate interventions, it is necessary to conduct more research on the dynamics of child undernutrition in high-burden settings.

In many cases, a malnourished child exhibits multiple indicators of undernutrition, often due to shared social disadvantages that contribute to several health deficiencies (14–16). This provides an opportunity to study multiple nutrition indicators simultaneously (17). Previous research has primarily focused on identifying the risk factors associated with child undernutrition using a single anthropometric indicator (18–20). This study employs a trivariate binary logistic regression analysis to examine the common risk factors for joint stunting, wasting, and underweight among children in Malawi.

The child undernutrition indicators are often paired; however, most public health research analyses them separately due to limitations in available statistical methods. Many studies focus on individual risk factors for stunting, underweight, and wasting, rather than looking at these indicators together. This research addresses that gap by analyzing the nutrition indicators jointly and identifying common risk factors for any two indicators simultaneously. This comprehensive analysis will enable policymakers to take decisive action based on the identified drivers of child undernutrition.

This study is organized as follows: Section 2 presents the data and statistical methods used, and Section 3 gives the results. This is followed by the discussion of findings in Section 4 and the conclusion in Section 5.

## 2 Methods

### 2.1 Data

The research utilized data from the household member recode file of the 2024 Malawi Demographic and Health Survey (MDHS), which aimed at collecting an up-to-date estimates regarding the basic demographic and health indicators in Malawi. The survey was implemented between 12 May and 31 August 2024 by the National Statistical Office (NSO) of Malawi, with the technical support from the ICF and DHS programme of the United States of America. The survey involved a two-stage cluster-stratified sampling technique in which 792 clusters were randomly sampled from across the country in the first stage and 23,070 households at second stage (21). A subsample of these households produced children's data reported by biological mothers, which had a total sample of 4,231 children aged 0–59 months. The interest of this study was to analyze joint undernutrition outcomes in the study area including clusters with extreme values. Some children had missing values in the response variables. This study has dropped the missing values and used complete case analysis, which involved a sample of 3,323 children.

The study involved three dependent variables. The first was whether or not the under-five child was stunted, which was characterized by height-for-age standardized score (HAZ) of less than or equal to –2.0. The second variable was whether the under-five child was underweight using weight-for-age standardized score (WAZ) of less than or equal to –2.0. The third variable was whether the under-five child was wasted, based on weight-for-height standardized score (WHZ) of less than or equal to –2.0 (7). Each child had measurements on these variables that were taken around the same time; hence, a joint or trivariate regression analysis was carried out. The independent variables included the child's sex, age in months, birth order number, preceding birth interval in months, and whether the child had malaria diagnosed by rapid test at the time of the survey (2, 12, 22). In addition, the mother's education level, place of residence, and household's wealth index were also included in the modeling (10, 11).

The 2024 MDHS data analyzed during this research are publicly available for users through the following link: https://www.dhsprogram.com/data/dataset/Malawi_Standard-DHS_2024.cfm?flag=1. Data cleaning was carried out using the STATA package version 12.0.

### 2.2 Trivariate logistic regression model and its estimation

Let (*Y*_*i*1_, *Y*_*i*2_, *Y*_*i*3_) represents number of observed cases of joint stunting, underweight, and wasting in *i*-th child surveyed. Let the actual measurements be represented by (*y*_*i*1_, *y*_*i*2_, *y*_*i*3_), where *i* = 1, 2, ..., *n* is the unit of analysis. At each observation, let 1 represents the outcome that a child was stunted, or underweight, or wasted, and 0 otherwise. This gave rise to a trivariate Bernoulli experiment with the following ordered triple outcome:


$$\begin{array}{l} \left(y_{i1},y_{i2},y_{i3}\right)=\{\left(0,0,0\right),\left(0,0,1\right),\left(0,1,0\right),\left(1,0,0\right),\\ \;\left(0,1,1\right),\left(1,0,1\right),\left(1,1,0\right),\left(1,1,1)\right\}, \end{array}$$ (1)

each event in Equation (1) occurring with respective probability:


$$\begin{array}{l} \begin{array}{c} p_{000}=P\left(Y_{i1}=0,Y_{i2}=0,Y_{i3}=0\right);\\ p_{001}=P\left(Y_{i1}=0,Y_{i2}=0,Y_{i3}=1\right);\\ p_{010}=P\left(Y_{i1}=0,Y_{i2}=1,Y_{i3}=0\right);\\ p_{100}=P\left(Y_{i1}=1,Y_{i2}=0,Y_{i3}=0\right);\\ p_{011}=P\left(Y_{i1}=0,Y_{i2}=1,Y_{i3}=1\right);\\ p_{101}=P\left(Y_{i1}=1,Y_{i2}=0,Y_{i3}=1\right);\\ p_{110}=P\left(Y_{i1}=1,Y_{i2}=1,Y_{i3}=0\right);\\ p_{111}=P\left(Y_{i1}=1,Y_{i2}=1,Y_{i3}=1\right), \end{array} \end{array}$$ (2)

where *p*_000_+*p*_001_+*p*_010_+*p*_100_+*p*_011_+*p*_101_+*p*_110_+*p*_111_ = 1. If the probabilities of events of 1's in Equation (2) are fixed and identical across the observations, then the set (*Y*_*i*1_, *Y*_*i*2_, *Y*_*i*3_) has a trivariate Bernoulli probability distribution, with the following mass function (23, 24):


$$\begin{array}{l} \begin{array}{l} p\left(y_{1},y_{2},y_{3}\right)=p_{111}^{y_{1}y_{2}y_{3}}p_{110}^{y_{1}y_{2}\left(1-y_{3}\right)}p_{101}^{y_{1}y_{3}\left(1-y_{2}\right)}p_{011}^{y_{2}y_{3}\left(1-y_{1}\right)}p_{100}^{y_{1}\left(1-y_{2}\right)\left(1-y_{3}\right)}\\ \;p_{010}^{y_{2}\left(1-y_{1}\right)\left(1-y_{3}\right)}p_{001}^{y_{3}\left(1-y_{1}\right)\left(1-y_{3}\right)}p_{000}^{\left(1-y_{1}\right)\left(1-y_{2}\right)\left(1-y_{3}\right)}\\ \;=\exp(y_{1}\log\left(\frac{p_{100}}{p_{000}}\right)+y_{2}\log\left(\frac{p_{010}}{p_{000}}\right)+y_{3}\log\left(\frac{p_{001}}{p_{000}}\right)\\ \;+y_{1}y_{2}\log\left(\frac{p_{110}p_{000}}{p_{100}p_{010}}\right)+y_{1}y_{3}\log\left(\frac{p_{101}p_{000}}{p_{100}p_{001}}\right)\\ \;+y_{2}y_{3}\log\left(\frac{p_{011}p_{000}}{p_{010}p_{001}}\right)\\ \;+y_{1}y_{2}y_{3}\log\left(\frac{p_{111}p_{100}p_{010}p_{001}}{p_{110}p_{101}p_{011}p_{000}}\right)+\log p_{000}). \end{array} \end{array}$$ (3)

As it can be appreciated from the exponential form of the trivariate Bernoulli distribution in Equation 3, the marginal and joint event probabilities of the three variables given in Equation 2 can be estimated though seven log odds natural parameters in a trivariate logistic model (23, 24). The trivariate logistic regression is given by


$$\begin{array}{l} \begin{array}{r} Y_{i1}=p_{100}\left(X\right)+\epsilon_{i1},\\ Y_{i2}=p_{010}\left(X\right)+\epsilon_{i2},\\ Y_{i3}=p_{001}\left(X\right)+\epsilon_{i3},\\ \left(Y_{i1},Y_{i2}\right)=p_{110}\left(X\right)+\epsilon_{i12},\\ \left(Y_{i1},Y_{i3}\right)=p_{101}\left(X\right)+\epsilon_{i13},\\ \left(Y_{i2},Y_{i3}\right)=p_{011}\left(X\right)+\epsilon_{i23},\\ \left(Y_{i1},Y_{i2},Y_{i3}\right)=p_{111}\left(X\right)+\epsilon_{i123}. \end{array} \end{array}$$ (4)

The probabilities in Equation (4) were estimated from the log odds link functions given below:


$$\begin{array}{l} \begin{array}{r} \log\left(\frac{p_{100}}{p_{000}}\right)=X_{i1}^{T}\beta ,\\ \log\left(\frac{p_{010}}{p_{000}}\right)=X_{i2}^{T}\beta ,\\ \log\left(\frac{p_{001}}{p_{000}}\right)=X_{i3}^{T}\beta ,\\ \log\left(\frac{p_{110}p_{000}}{p_{100}p_{010}}\right)=X_{i12}^{T}\beta ,\\ \log\left(\frac{p_{101}p_{000}}{p_{100}p_{001}}\right)=X_{i13}^{T}\beta ,\\ \log\left(\frac{p_{011}p_{000}}{p_{010}p_{001}}\right)=X_{i23}^{T}\beta ,\\ \log\left(\frac{p_{111}p_{100}p_{010}p_{001}}{p_{110}p_{101}p_{011}p_{000}}\right)=X_{i123}^{T}\beta , \end{array} \end{array}$$ (5)

where $X_{iw}^{T}=\left(1,X_{i1},X_{i2},...,X_{ip}\right)$ is vector of independent variables' values observed on *i*-th child; $\beta ={\left(\beta_{0},\beta_{1},\beta_{2},...,\beta_{p}\right)}^{T}$ were the fixed effects, while ϵ_*i*_ represented the model's error term at each level of the response variable, which was assumed to have mean zero (25).

Solving the system of equations in Equation 5 provided the following estimates of the probabilities given in model Equation 4


$$\begin{array}{l} \begin{array}{l} p_{100}=p_{000}\exp\left(X_{i1}^{T}\beta \right),\\ p_{010}=p_{000}\exp\left(X_{i2}^{T}\beta \right),\\ p_{001}=p_{000}\exp\left(X_{i3}^{T}\beta \right),\\ p_{110}=p_{000}\exp\left(X_{i1}^{T}\beta +X_{i2}^{T}\beta +X_{i12}^{T}\beta \right),\\ p_{101}=p_{000}\exp\left(X_{i1}^{T}\beta +X_{i3}^{T}\beta +X_{i13}^{T}\beta \right),\\ p_{011}=p_{000}\exp\left(X_{i2}^{T}\beta +X_{i3}^{T}\beta +X_{i23}^{T}\beta \right),\\ p_{111}=p_{000}\exp(X_{i1}^{T}\beta +X_{i2}^{T}\beta +X_{i3}^{T}\beta +X_{i12}^{T}\beta \\ \;+X_{i13}^{T}\beta +X_{i23}^{T}\beta +X_{i123}^{T}\beta ), \end{array} \end{array}$$ (6)

where the probability *p*_000_ in Equation (6) is given by Dai (23) and Islam (24):


$$\begin{array}{l} \begin{array}{l} p_{000}=\frac{1}{A},\\ \;A=1+\exp\left(X_{i1}^{T}\beta \right)+\exp\left(X_{i2}^{T}\beta \right)+\exp\left(X_{i3}^{T}\beta \right)\\ \;+\exp\left(X_{i1}^{T}\beta +X_{i2}^{T}\beta +X_{i12}^{T}\beta \right)\\ \;+\exp\left(X_{i1}^{T}\beta +X_{i3}^{T}\beta +X_{i13}^{T}\beta \right)\\ \;+\exp\left(X_{i2}^{T}\beta +X_{i3}^{T}\beta +X_{i23}^{T}\beta \right)\\ \;+\exp(X_{i1}^{T}\beta +X_{i2}^{T}\beta +X_{i3}^{T}\beta +X_{i12}^{T}\beta \\ \;+X_{i13}^{T}\beta +X_{i23}^{T}\beta +X_{i123}^{T}\beta ). \end{array} \end{array}$$ (7)

The model parameters were estimated using maximum likelihood given by


$$\begin{array}{l} L\left(\beta ,\sigma_{b}^{2}\right)=\overset{n}{\underset{i=1}{\prod }}\exp(y_{1}\left(X_{i1}^{T}\beta \right)+y_{2}\left(X_{i2}^{T}\beta \right)+y_{3}\left(X_{i3}^{T}\beta \right)\\ \;+y_{1}y_{2}\left(X_{i12}^{T}\beta \right)+y_{1}y_{3}\left(X_{i13}^{T}\beta \right)+y_{2}y_{3}\left(X_{i23}^{T}\beta \right)\\ \;+y_{1}y_{2}y_{3}\left(X_{i123}^{T}\beta \right)-\log A), \end{array}$$ (8)

where *A* is as defined in Equation 7. The partial derivatives of the log-likelihood function derived from Equation (8) with respect to the model fixed parameters pertinent to particular marginal model provided the score functions (26), which helped to find the model solutions. The random effects were estimated in turn, using the updated log-likelihood function. The fixed effect ML estimates $\hat{\beta }$ have usual interpretation as marginal log odds of success in the respective outcome, when comparing one level of a covariate to the other called reference level. In this research, the interest was on estimating risk factors of joint occurrence of stunting and underweight, stunting and wasting, as well and underweight and wasting. The joint model estimates were calculated using the R package VGAM (27) in R software version 4.5.3. The R codes used are given as an Appendix.

## 3 Results

### 3.1 Data summary

The summary of the data given in Table 1 showed that cases of low height-for-age (i.e., stunting), low weight-for-age (i.e., underweight), and weight-for-height (i.e., wasting) were present in less than 5% of the studied children. The conditions were generally more prevalent in male children, those aged 12 months and above, those staying in poor households, and those with preceding birth interval of not more than 36 months. In addition, three conditions were also in large proportions in children who breastfed 13 months and above, those whose mothers had no education or primary education, and children residing in rural areas. The chi-squared test results showed that stunting was significantly associated with a child's sex and preceding birth interval. While underweight was associated with the child's age group, his or her preceding birth interval, duration of breastfeeding, mother's education, household's wealth, and place of residence, wasting was associated with the child's age group, and his or her duration of breastfeeding.

**Table 1:** Distribution of unweighted cases of stunting, underweight, and wasting by the socio-demographic characteristics of the children, 2024 MDHS complete cases data.

	Stunting			Underweight		Wasting	
Characteristic	*n* (%)	Stunted (%)	χ^2^ p-value	Underweight (%)	χ^2^ *p*-value	Wasted (%)	χ^2^ *p*-value
Overall sample	5,370 (100)	105 (01.96)		125 (02.33)		128 (02.38)	
Child's sex			0.029		0.220		0.166
Male	2,654 (49.42)	63 (02.37)		55 (02.07)		71 (02.68)	
Female	2,716 (50.58)	42 (01.55)		70 (02.58)		57 (02.10)	
Child's age group			0.308		0.011		0.001
0–11 months	1,160 (21.60)	17 (01.47)		13 (01.12)		19 (01.64)	
12–23 months	1,170 (21.79)	21 (01.79)		28 (02.39)		46 (03.93)	
24–35 months	1,028 (19.4)	22 (02.14)		35 (03.40)		27 (02.63)	
36–47 months	1,002 (18.66)	27 (02.69)		23 (02.30)		21 (02.10)	
48–59 months	1,010 (18.81)	18 (01.78)		26 (02.58)		15 (01.49)	
Preceding birth interval			< 0.001		0.069		0.934
< 24 months	291 (05.42)	9 (03.09)		10 (03.44)		6 (02.06)	
24–36 months	628 (11.69)	26 (04.14)		21 (03.34)		15 (02.39)	
>36 months	4,451 (82.89)	70 (01.57)		94 (02.11)		107 (02.4)	
Malaria result			0.292		0.877		0.427
Negative	3,472 (64.66)	73 (02.10)		80 (02.30)		87 (02.51)	
Positive	1,898 (35.34)	32 (01.69)		45 (02.37)		41 (02.16)	
Mother's education			0.415		0.056		0.800
No education	376 (07.00)	9 (02.39)		12 (03.19)		9 (02.39)	
Primary	3,489 (64.97)	74 (02.12)		91 (02.61)		88 (02.52)	
Secondary	1,393 (25.94)	20 (01.44)		21 (01.51)		29 (02.08)	
Higher	112 (02.09)	2 (01.79)		1 (00.89)		2 (01.79)	
Household's wealth			0.104		< 0.001		0.820
Poor	2,371 (44.15)	57 (02.40)		79 (03.33)		59 (02.49)	
Middle	883 (16.44)	15 (01.70)		11 (01.25)		22 (02.49)	
Rich	2,116 (39.4)	33 (01.56)		35 (01.65)		47 (02.22)	
Place of residence			0.447		0.004		0.838
Urban	970 (18.06)	16 (01.65)		16 (01.65)		24 (02.47)	
Rural	4,400 (81.94)	89 (02.02)		109 (02.48)		104 (02.36)	
Mean birthorder (SD)	2.85 (01.93)						

SD = standard deviation, χ^2^ = chi-squared test, *n* = sample size.

### 3.2 Effects of socio-demographic factors on joint child stunting and underweight, and wasting based on the trivariate logistic regression model

The model estimates presented in Table 2 indicated that the log odds of being both stunted and underweight were significantly lower in children aged 12 months and older, those with preceding birth interval of 24 months and above, children from middle income or rich families, and those residing in rural areas. The log odds of both stunting and underweight increased with increasing birth order. While the effects of being malarial, being female, and mother's education were not significant, the estimate of model intercept showed that cases of joint stunting and underweight were on the rise in the study population.

**Table 2:** Effects of child characteristics on joint stunting and underweight upon fitting a bivariate logistic regression model to 2024MDHS data.

Variable	Stunting Log odds (SE, *p*-value)	Underweight Log odds (SE, *p*-value)	Stunting and underweight Log odds (SE, *p*-value)
Intercept	–1.51 (1.29, 0.2439)	–3.27 (1.29, 0.0114)	28.5 (12.4, 0.0218)
Child's sex			
Male*			
Female	–0.32 (0.29, 0.2689)	0.13 (0.27, 0.6236)	1.43 (1.83, 0.4344)
Child's age group			
0–11 months*			
12–23 months	–0.26 (0.49, 0.5921)	–0.03 (0.48, 0.9565)	–13.0 (5.87, 0.0265)
24–35 months	–0.13 (0.44, 0.7651)	0.49 (0.42, 0.2396)	–7.50 (3.88, 0.0531)
36–47 months	–0.31 (0.49, 0.5305)	–0.02 (0.48, 0.9626)	–12.2 (5.75, 0.0334)
48–59 months	–0.12 (0.45, 0.7967)	0.22 (0.44, 0.6245)	–3.77 (3.28, 0.2505)
Preceding birth interval			
< 24 months*			
24–36 months	0.39 (0.44, 0.3761)	0.54 (0.46, 0.2411)	–4.40 (3.27, 0.1790)
>36 months	–1.02 (0.44, 0.0204)	–0.12 (0.44, 0.7802)	–10.6 (5.09, 0.0368)
Malaria result			
Negative*			
Positive	–0.01 (0.36, 0.9866)	0.03 (0.33, 0.9366)	–0.68 (2.18, 0.7535)
Mother's education			
No education*			
Primary	–0.46 (0.46, 0.3174)	–0.04 (0.44, 0.9342)	–4.11 (3.23, 0.2038)
Secondary	–0.29 (0.60, 0.6277)	–0.78 (0.63, 0.2167)	6.09 (4.41, 0.1674)
Higher	0.89 (0.98, 0.3622)	0.57 (1.21, 0.6382)	–11.1 (8.28, 0.1817)
Household's wealth			
Poor*			
Middle	–0.56 (0.49, 0.2526)	–1.51 (0.56, 0.0067)	–6.97 (4.75, 0.1421)
Rich	–0.61 (0.41, 0.1378)	–0.99 (0.40, 0.0132)	–7.66 (4.14, 0.0642)
Place of residence			
Urban*			
Rural	–0.31 (0.49, 0.5310)	–0.32 (0.49, 0.5191)	–10.3 (4.89, 0.0354)
Birthorder	–0.03 (0.10, 0.7629)	0.09 (0.08, 0.3092)	3.35 (1.46, 0.0223)

SE, standard error; (*), reference category.

In the marginal univariate outcomes, the log odds of stunting were found to be significantly lower in children with birth interval of above 36 months, while the log odds of underweight were lower in children from middle and rich wealth quintile households. The p-values indicated that most variables had significant effects in the paired outcome compared with the marginal univariate outcomes. However, the directions of the estimates for most of the child characteristics were consistent between the bivariate and univariate outcomes.

The estimates given in Table 3 are for effects of child characteristics on joint stunting and wasting, as well as on their marginal outcomes. The results showed that the log odds of being both stunted and wasted were lower in children aged 12 months and older, and those residing in rural areas, although the effects were not statistically significant. None of the variables has neither significant effects in univariate outcome case.

**Table 3:** Effects of child characteristics on joint stunting and wasting upon fitting a bivariate logistic regression model to 2024MDHS data.

	Stunting	Wasting	Stunting and wasting
Variable	Log odds (SE, *p*-value)	Log odds (SE, *p*-value)	Log odds (SE, *p*-value)
Intercept	-1.83 (2.11, 0.3859)	-4.08 (2.13, 0.0555)	-48.1 (38.9, 0.2163)
Child's sex			
Male*			
Female	–0.30 (0.47, 0.5284)	-0.40 (0.40, 0.3169)	11.5 (8.07, 0.1526)
Child's age group			
0–11 months*			
12–23 months	–0.07 (0.54, 0.8983)	0.63 (0.44, 0.1512)	–0.34 (3.90, 0.9312)
24–35 months	–0.09 (0.61, 0.8894)	0.18 (0.54, 0.7365)	–5.84 (6.09, 0.3377)
36–47 months	0.01 (2.18, 0.9952)	0.05 (2.17, 0.9799)	–26.7 (28.4, 0.3484)
48–59 months	–0.05 (0.69, 0.9390)	–0.24 (0.68, 0.7178)	–9.40 (7.98, 0.2390)
Preceding birth interval			
< 24 months*			
24–36 months	0.33 (0.94, 0.7280)	0.74 (1.36, 0.5868)	5.62 (10.0, 0.5757)
>36 months	–0.87 (0.94, 0.3566)	0.80 (1.31, 0.5404)	14.6 (13.2, 0.2689)
Malaria result			
Negative*			
Positive	–0.20 (0.70, 0.7724)	–0.94 (0.67, 0.1576)	17.7 (12.9, 0.1723)
Mother's education			
No education*			
Primary	–0.48 (0.72, 0.5007)	0.02 (0.63, 0.9711)	2.19 (6.17, 0.7225)
Secondary	–0.39 (0.82, 0.6365)	–0.40 (0.74, 0.5857)	11.1 (10.3, 0.2835)
Higher	0.70 (1.36, 0.6071)	0.27 (1.55, 0.8637)	–6.05 (16.7, 0.7168)
Household's wealth			
Poor*			
Middle	–0.68 (0.78, 0.3836)	–0.08 (0.55, 0.8902)	8.58 (8.16, 0.2926)
Rich	–0.41 (0.62, 0.5070)	–0.33 (0.52, 0.5209)	12.8 (9.83, 0.1936)
Place of residence			
Urban*			
Rural	–0.19 (0.53, 0.7171)	0.12 (0.48, 0.7983)	–5.99 (5.56, 0.2812)
Birthorder	–0.03 (0.13, 0.7957)	0.04 (0.11, 0.7042)	3.41 (2.61, 0.1910)

SE, standard error; (*), reference category.

The results in Table 4 showed that the log odds of being both underweight and wasted were lower in children living in middle income households. The log odds of both underweight and wasting were higher in children aged 12–47 months. In the marginal univariate outcomes, the log odds of underweight were significantly lower in children from middle wealth family, while chances of wasting were low in malarial children and high in children aged 12–23 months. Again, most variables had no significant effects on either underweight or wasting or both.

**Table 4:** Effects of child characteristics on joint underweight and wasting upon fitting a bivariate logistic regression model to 2024MDHS data.

Variable	Underweight Log odds (SE, *p*-value)	Wasting Log odds (SE, *p*-value)	Underweight and wasting Log odds (SE, *p*-value)
Intercept	–3.34 (1.92, 0.0818)	–4.50 (1.98, 0.0230)	–41.0 (219.2, 0.8517)
Child's sex			
Male*			
Female	0.18 (0.30, 0.5503)	–0.31 (0.29, 0.2834)	–1.22 (1.40, 0.3832)
Child's age group			
0–11 months*			
12–23 months	0.35 (0.51, 0.4894)	0.82 (0.45, 0.0691)	4.26 (2.38, 0.0734)
24–35 months	0.64 (0.48, 0.1852)	0.39 (0.48, 0.4188)	2.88 (2.03, 0.1550)
36–47 months	0.31 (0.51, 0.5429)	0.39 (0.49, 0.4198)	2.72 (2.13, 0.2024)
48–59 months	0.24 (0.53, 0.6485)	–0.02 (0.56, 0.9698)	–10.6 (109.2, 0.9226)
Preceding birth interval			
< 24 months*			
24–36 months	0.31 (0.56, 0.5762)	0.50 (0.73, 0.4940)	0.20 (2.59, 0.9374)
>36 months	–0.22 (0.51, 0.6602)	0.69 (0.65, 0.2944)	3.76 (2.55, 0.1400)
Malaria result			
Negative*			
Positive	0.13 (0.37, 0.7145)	–1.13 (0.54, 0.0371)	–0.47 (2.07, 0.8203)
Mother's education			
No education*			
Primary	–0.16 (0.45, 0.7218)	0.09 (0.51, 0.8626)	–0.07 (0.39, 0.858)
Secondary	–1.11 (1.38, 0.4221)	–0.43 (1.05, 0.6815)	–18.0 (109.6, 0.8692)
Higher	0.11 (4.01, 0.9775)	0.04 (4.71, 0.9926)	24.6 (275.0, 0.9286)
Household's wealth			
Poor*			
Middle	–1.28 (0.64, 0.0466)	–0.18 (0.41, 0.6540)	–5.74 (3.32, 0.0835)
Rich	–0.91 (0.71, 0.2009)	–0.41 (0.73, 0.5749)	19.4(109.5, 0.8597)
Place of residence			
Urban*			
Rural	–0.30 (0.82, 0.7105)	0.33 (0.83, 0.6906)	18.1 (109.5, 0.8684)
Birthorder	0.08 (0.07, 0.2329)	0.01 (0.07, 0.9122)	0.22 (0.33, 0.5109)

SE, standard error; (*), reference category.

## 4 Discussion

This article investigated the risks of multiple undernutrition conditions in a under-five child in Malawi. This was analyzed using a trivariate logistic regression model applied to the 2024 Malawi demographic and health survey data. The findings revealed strong evidence of risk of concurrent stunting and underweight in a child, but weak evidence of joint stunting and wasting, as well as underweight and wasting risks. Cases of paired stunting and underweight were explained by more independent variables involved compared to those of stunting and wasting, or underweight and wasting. Multiple child undernutrition outcomes in a child were expected, as the conditions relate to food intake in the child, making it unlikely for a patient to have one condition only and not the other (14, 28, 29).

The likelihood of joint stunting and underweight in children was low among children aged 12 months and older, those with preceding birth interval of 24 months and above, children from middle income or rich families, and those residing in rural areas. After breastfeeding cessation, older children in developing countries often experience periods of nutritional deprivation (30), which adversely affects their growth and development. Thus, the observed low risk of stunting and underweight in children over 12 months old is unexpected result. A long and adequate preceding birth interval allows the mother to provide better healthcare, attention, and nutrition for the youngest child at home, which may likely reduce the chances of stunting and underweight in that child (31). This also enables proper adherence to exclusive and adequate breastfeeding and hence reducing chances of stunting and underweight (32, 33). Children from wealthier households are food secure, which is why they have low chances of being stunted and underweight (34).

Furthermore, children living in rural areas usually have access to affordable food as Malawi's economy is agrarian and heavily supported by the rural population (35). So, it was not surprising in this study to observe low chances of joint stunting and underweight in children residing in rural areas. The findings also indicated that the chances of stunting and underweight increased with increasing birth order. Higher birth order is associated with thinness, probably due to dampened maternal excitement and attention which might reduce dietary care that benefits first-born and second-born children (2, 13, 36).

The study had approximately one-fifth of the sample with missing data on both outcome and explanatory variables, and the corresponding cases were dropped to have a complete case analysis. This reduced the sample size and might have biased the estimates of standard errors and p-values in this study (37). The VGAM package of R software that was used to process the data could only produce estimates up to two paired outcomes, and not three as planned (27). This limited the scope of the estimations in this study, despite availability of statistical methods.

## 5 Conclusion

This research presents evidence of common risk factors and the simultaneous occurrence of child undernutrition indicators, specifically low height-for-age (stunting) and low weight-for-age (underweight). It emphasizes the necessity of using statistical methods that can analyse these indicators simultaneously. By applying a trivariate logistic regression to the 2024 Malawi Demographic and Health Survey data, the study examined common risk factors associated with concurrent stunting and underweight, stunting and wasting, and underweight and wasting. It was found that the risk of stunting and underweight was low in children older than 12 months, those from wealthier families, and those who resided in rural areas. The risk was higher in children with higher birth order. The risk of joint stunting and wasting or underweight and wasting could not be much explained by the variables involved in the modeling. Given that health outcomes can be paired for an individual child, this study recommends utilizing statistical methods that analyze these outcomes simultaneously rather than sequentially. This approach has the potential to reveal common risk factors related to multiple health issues, facilitating the development of health policies that address all related concerns collectively. Future research could combine these multivariate regression methods with techniques designed to handle missing data.

## Data Availability

Publicly available datasets were analyzed in this study. This data can be found here: https://www.dhsprogram.com/data/dataset/Malawi_Standard-DHS_2024.cfm?flag=1.
