# Supplementary material for: Concurrent child undernutrition indicators in Malawi: application of trivariate logistic regression
Source: Front Nutr. 2026 May 29;13:1848833. doi: 10.3389/fnut.2026.1848833 (PMC13261811; doi:10.3389/fnut.2026.1848833)
Supplement: Supplementary file 1 [file Data_Sheet_1.pdf]

**Appendix: Software codes used for data management and analysis**

**STATA codes used for data handling (complete case) in the joint child under-nutrition study for 2024 MDHS data**

```
drop if hc70==9996
drop if hc70==9998 //dropping missing values for hc70
drop if hc70==9999
drop if hc70==.
Drop if hc70==.a
summarize hc70
gen HAZ = (hc70 -   -153.2628)/135.4289 //standardize hc70
gen Stunted=.
replace Stunted=1 if HAZ <= -2
replace Stunted=0 if HAZ > -2
label variable Stunted "child stunted"

drop if hc71==9996
drop if hc71==9998
drop if hc71==9999
drop if hc71==.
drop if hc71==.a
summarize hc71
gen WAZ = (hc71 -  -63.47263)/108.6166 //standardize hc71
gen Underweight=.
replace Underweight=1 if WAZ <= -2
replace Underweight=0 if WAZ > -2
label variable Underweight "child underweight"

drop if hc72==9996
drop if hc72==9998
drop if hc72==9999
drop if hc72==.
drop if hc72==.a
summarize hc72
```

```
gen WHZ=(hc72 - 33.6087)/116.1762
gen Wasted=.
replace Wasted=1 if WHZ <= -2
replace Wasted=0 if WHZ > -2
label variable Wasted "child wasted"

gen Sex = hc27

gen Birthorder = hc64

gen ChildAge = hc1
recode ChildAge 0/11=1
recode ChildAge 12/23=2
recode ChildAge 24/35=3
recode ChildAge 36/47=4
recode ChildAge 48/59=5
label define ChildAge 1"0-11 months" 2"12-23 months" 3"24-35 months" 4"36-47
months" 5"48-59 months"
label values ChildAge Childage

drop if hc63==999
gen PBInterval=hc63
recode PBInterval 0/23=1
recode PBInterval 23/36=2
recode PBInterval 37/269=3
label define PBInterval 1"< 24 months" 2"24-36 months" 3"> 36 months"
label values PBInterval PBInterval

gen Malaria=hml35
recode Malaria 3=0
recode Malaria 6=0
recode Malaria .=0

gen Education=hc61
gen residence = hv025
```

```
gen Wealth = hv270
recode Wealth 1/2=1
recode Wealth 3=2
recode Wealth 4/5=3
label define Wealth 1"poor" 2"middle" 3"rich"
label values Wealth Wealth
```

**R codes for fitting bivariate or trivariate logistic regression model to 2024 MDHS data**

```
rm(list=ls())
library(foreign)
library("VGAMdata")
library("bbmle")
library(dplyr)
library(data.table)
library(readstata13)

data = read.dta("C:/Users/User/Desktop/Nutrition
paper/MWPR_ed.dta",convert.factors=F)

model1 <- vglm(cbind(Stunted,Underweight) ~ Sex + residence + Birthorder + as
.factor(PBInterval) + as.factor(Education)
               +Malaria + as.factor(Wealth) + as.factor(ChildAge) ,
               binom2.or(exchangeable = FALSE,zero=NULL,tol = 0.001), data =
data, trace=T)

> summary(model1)

Call:
vglm(formula = cbind(Stunted, Underweight) ~ Sex + residence +
    Birthorder + as.factor(PBInterval) + as.factor(Education) +
    Malaria + as.factor(Wealth) + as.factor(ChildAge), family = binom2.or(exc
hangeable = FALSE,
    zero = NULL, tol = 0.001), data = data, trace = T)

Coefficients:
                           Estimate Std. Error z value Pr(>|z|)
(Intercept):1             -1.508167   1.294253  -1.165  0.24391
(Intercept):2             -3.266005   1.290856  -2.530  0.01140 *
(Intercept):3             28.471888  12.415248   2.293  0.02183 *
Sex:1                     -0.320582   0.289948  -1.106  0.26888
```

```
Sex:2                        0.133047   0.271114   0.491  0.62361
Sex:3                        1.426881   1.825203   0.782  0.43435
residence:1                 -0.306023   0.488463  -0.627  0.53099
residence:2                 -0.316045   0.490203  -0.645  0.51911
residence:3                -10.288686   4.891774  -2.103  0.03544 *
Birthorder:1                -0.029129   0.096572  -0.302  0.76293
Birthorder:2                 0.086193   0.084766   1.017  0.30924
Birthorder:3                 3.345699   1.464104   2.285  0.02230 *
as.factor(PBInterval)2:1     0.388048   0.438505   0.885  0.37619
as.factor(PBInterval)2:2     0.535362   0.456696   1.172  0.24110
as.factor(PBInterval)2:3    -4.400582   3.274560  -1.344  0.17899
as.factor(PBInterval)3:1    -1.022369   0.440888  -2.319  0.02040 *
as.factor(PBInterval)3:2    -0.123252   0.441634  -0.279  0.78018
as.factor(PBInterval)3:3   -10.633640   5.091435  -2.089  0.03675 *
as.factor(Education)1:1     -0.464252   0.464307  -1.000  0.31737
as.factor(Education)1:2     -0.036613   0.443732  -0.083  0.93424
as.factor(Education)1:3     -4.108732   3.233405  -1.271  0.20383
as.factor(Education)2:1     -0.291078   0.600107  -0.485  0.62765
as.factor(Education)2:2     -0.782032   0.633060  -1.235  0.21671
as.factor(Education)2:3      6.094955   4.414774   1.381  0.16741
as.factor(Education)3:1      0.893300   0.980306   0.911  0.36217
as.factor(Education)3:2      0.568655   1.209466   0.470  0.63823
as.factor(Education)3:3    -11.050736   8.275196  -1.335  0.18174
Malaria:1                   -0.006009   0.357617  -0.017  0.98659
Malaria:2                    0.026437   0.332375   0.080  0.93660
Malaria:3                   -0.684181   2.178357  -0.314  0.75346
as.factor(Wealth)2:1        -0.557894   0.487684  -1.144  0.25264
as.factor(Wealth)2:2        -1.510564   0.557198  -2.711  0.00671 **
as.factor(Wealth)2:3        -6.968027   4.746614  -1.468  0.14210
as.factor(Wealth)3:1        -0.610843   0.411577  -1.484  0.13777
as.factor(Wealth)3:2        -0.992059   0.400266  -2.478  0.01319 *
as.factor(Wealth)3:3        -7.656310   4.137016  -1.851  0.06422 .
as.factor(ChildAge)2:1      -0.262030   0.489034  -0.536  0.59209
as.factor(ChildAge)2:2      -0.026023   0.477015  -0.055  0.95649
as.factor(ChildAge)2:3     -13.022996   5.867523  -2.220  0.02645 *
as.factor(ChildAge)3:1      -0.131954   0.441617  -0.299  0.76509
as.factor(ChildAge)3:2       0.489289   0.416086   1.176  0.23962
as.factor(ChildAge)3:3      -7.495854   3.875742  -1.934  0.05311 .
as.factor(ChildAge)4:1      -0.308601   0.492019  -0.627  0.53052
as.factor(ChildAge)4:2      -0.022680   0.483069  -0.047  0.96255
as.factor(ChildAge)4:3     -12.241824   5.754451  -2.127  0.03339 *
as.factor(ChildAge)5:1      -0.116316   0.451490  -0.258  0.79669
as.factor(ChildAge)5:2       0.215787   0.440893   0.489  0.62454
as.factor(ChildAge)5:3      -3.769988   3.280646  -1.149  0.25049
---
Signif. codes:  0 ‘***’ 0.001 ‘**’ 0.01 ‘*’ 0.05 ‘.’ 0.1 ‘ ’ 1

Names of linear predictors: logitlink(mu1), logitlink(mu2), loglink(oratio)

Residual deviance: 786.5761 on 7014 degrees of freedom

Log-likelihood: NA on 7014 degrees of freedom

Number of Fisher scoring iterations: 30
```

```
model2 <- vglm(cbind(Stunted,Wasted) ~ Sex + residence + Birthorder + as.fact
or(PBInterval) + as.factor(Education)
               +Malaria + as.factor(Wealth) + as.factor(ChildAge) ,
               binom2.or(exchangeable = FALSE,zero=NULL,tol = 0.001), data =
data, trace=T)

> summary(model2)

Call:
vglm(formula = cbind(Stunted, Wasted) ~ Sex + residence + Birthorder +
    as.factor(PBInterval) + as.factor(Education) + Malaria +
    as.factor(Wealth) + as.factor(ChildAge), family = binom2.or(exchangeable
= FALSE,
    zero = NULL, tol = 0.001), data = data, trace = T)

Coefficients:
                          Estimate Std. Error z value Pr(>|z|)
(Intercept):1             -1.83010    2.11069  -0.867   0.3859
(Intercept):2             -4.07646    2.12920  -1.915   0.0555 .
(Intercept):3            -48.12453   38.91861  -1.237   0.2163
Sex:1                     -0.29946    0.47495  -0.631   0.5284
Sex:2                     -0.39835    0.39801  -1.001   0.3169
Sex:3                     11.54151    8.06850   1.430   0.1526
residence:1               -0.19163    0.52895  -0.362   0.7171
residence:2                0.12324    0.48220   0.256   0.7983
residence:3               -5.99463    5.56335  -1.078   0.2812
Birthorder:1              -0.03381    0.13062  -0.259   0.7957
Birthorder:2               0.04305    0.11337   0.380   0.7042
Birthorder:3               3.41343    2.61025   1.308   0.1910
as.factor(PBInterval)2:1   0.32566    0.93648   0.348   0.7280
as.factor(PBInterval)2:2   0.74094    1.36327   0.544   0.5868
as.factor(PBInterval)2:3   5.61858   10.03938   0.560   0.5757
as.factor(PBInterval)3:1  -0.86835    0.94192  -0.922   0.3566
as.factor(PBInterval)3:2   0.80324    1.31194   0.612   0.5404
as.factor(PBInterval)3:3  14.63552   13.23865   1.106   0.2689
as.factor(Education)1:1   -0.48272    0.71685  -0.673   0.5007
as.factor(Education)1:2    0.02283    0.63085   0.036   0.9711
as.factor(Education)1:3    2.18930    6.16511   0.355   0.7225
as.factor(Education)2:1   -0.38717    0.81935  -0.473   0.6365
as.factor(Education)2:2   -0.40473    0.74258  -0.545   0.5857
as.factor(Education)2:3   11.09058   10.34062   1.073   0.2835
as.factor(Education)3:1    0.69730    1.35610   0.514   0.6071
as.factor(Education)3:2    0.26635    1.55186   0.172   0.8637
as.factor(Education)3:3   -6.05402   16.68788  -0.363   0.7168
Malaria:1                 -0.20187    0.69796  -0.289   0.7724
Malaria:2                 -0.94475    0.66859  -1.413   0.1576
Malaria:3                 17.65815   12.93926   1.365   0.1723
as.factor(Wealth)2:1      -0.68175    0.78241  -0.871   0.3836
as.factor(Wealth)2:2      -0.07607    0.55104  -0.138   0.8902
```

```
as.factor(Wealth)2:3       8.58271    8.15525   1.052   0.2926
as.factor(Wealth)3:1      -0.41084    0.61913  -0.664   0.5070
as.factor(Wealth)3:2      -0.33318    0.51895  -0.642   0.5209
as.factor(Wealth)3:3      12.77925    9.83014   1.300   0.1936
as.factor(ChildAge)2:1    -0.06853    0.53637  -0.128   0.8983
as.factor(ChildAge)2:2     0.62950    0.43863   1.435   0.1512
as.factor(ChildAge)2:3    -0.33658    3.89780  -0.086   0.9312
as.factor(ChildAge)3:1    -0.08530    0.61355  -0.139   0.8894
as.factor(ChildAge)3:2     0.18317    0.54447   0.336   0.7365
as.factor(ChildAge)3:3    -5.83933    6.09054  -0.959   0.3377
as.factor(ChildAge)4:1     0.01300    2.17714   0.006   0.9952
as.factor(ChildAge)4:2     0.05455    2.16790   0.025   0.9799
as.factor(ChildAge)4:3   -26.65090   28.42083  -0.938   0.3484
as.factor(ChildAge)5:1    -0.05259    0.68666  -0.077   0.9390
as.factor(ChildAge)5:2    -0.24425    0.67590  -0.361   0.7178
as.factor(ChildAge)5:3    -9.39704    7.98061  -1.177   0.2390
---
Signif. codes:  0 ‘***’ 0.001 ‘**’ 0.01 ‘*’ 0.05 ‘.’ 0.1 ‘ ’ 1

Names of linear predictors: logitlink(mu1), logitlink(mu2), loglink(oratio)

Residual deviance: 916.2881 on 7014 degrees of freedom

Log-likelihood: NA on 7014 degrees of freedom

Number of Fisher scoring iterations: 30


model3 <- vglm(cbind(Underweight,Wasted) ~ Sex + residence + Birthorder + as.
factor(PBInterval) + as.factor(Education)
               +Malaria + as.factor(Wealth) + as.factor(ChildAge) ,
               binom2.or(exchangeable = FALSE,zero=NULL,tol = 0.001), data =
data, trace=T)

> summary(model3)

Call:
vglm(formula = cbind(Underweight, Wasted) ~ Sex + residence +
    Birthorder + as.factor(PBInterval) + as.factor(Education) +
    Malaria + as.factor(Wealth) + as.factor(ChildAge), family = binom2.or(exc
hangeable = FALSE,
    zero = NULL, tol = 0.001), data = data, trace = T)

Coefficients:
                           Estimate Std. Error z value Pr(>|z|)
(Intercept):1             -3.342447   1.920381  -1.741   0.0818 .
(Intercept):2             -4.503012   1.981208  -2.273   0.0230 *
(Intercept):3            -40.962020 219.152592  -0.187   0.8517
Sex:1                      0.177092   0.296464   0.597   0.5503
Sex:2                     -0.312568   0.291382  -1.073   0.2834
Sex:3                     -1.221790   1.401245  -0.872   0.3832
residence:1               -0.303865   0.818641  -0.371   0.7105
residence:2                0.330228   0.829531   0.398   0.6906
```

```
residence:3              18.146763 109.525990   0.166   0.8684
Birthorder:1              0.081761   0.068545   1.193   0.2329
Birthorder:2              0.008236   0.074660   0.110   0.9122
Birthorder:3              0.219180   0.333364   0.657   0.5109
as.factor(PBInterval)2:1   0.310833   0.556086   0.559   0.5762
as.factor(PBInterval)2:2   0.498293   0.728487   0.684   0.4940
as.factor(PBInterval)2:3   0.203294   2.588988   0.079   0.9374
as.factor(PBInterval)3:1  -0.224380   0.510353  -0.440   0.6602
as.factor(PBInterval)3:2   0.685645   0.653904   1.049   0.2944
as.factor(PBInterval)3:3   3.762461   2.549219   1.476   0.1400
as.factor(Education)1:1   -0.161012   0.452258  -0.356   0.7218
as.factor(Education)1:2    0.087443   0.505250   0.173   0.8626
as.factor(Education)1:3    5.090687   2.539497   2.005   0.0450 *
as.factor(Education)2:1   -1.106523   1.378388  -0.803   0.4221
as.factor(Education)2:2   -0.432305   1.053381  -0.410   0.6815
as.factor(Education)2:3  -18.049560 109.588808  -0.165   0.8692
as.factor(Education)3:1    0.112988   4.005674   0.028   0.9775
as.factor(Education)3:2    0.043782   4.714533   0.009   0.9926
as.factor(Education)3:3   24.646239 275.022058   0.090   0.9286
Malaria:1                 0.133915   0.366086   0.366   0.7145
Malaria:2                -1.126825   0.540661  -2.084   0.0371 *
Malaria:3                -0.469774   2.067624  -0.227   0.8203
as.factor(Wealth)2:1      -1.276393   0.641536  -1.990   0.0466 *
as.factor(Wealth)2:2      -0.184448   0.411481  -0.448   0.6540
as.factor(Wealth)2:3      -5.738711   3.315866  -1.731   0.0835 .
as.factor(Wealth)3:1      -0.912496   0.713423  -1.279   0.2009
as.factor(Wealth)3:2      -0.409306   0.729857  -0.561   0.5749
as.factor(Wealth)3:3      19.357224 109.510264   0.177   0.8597
as.factor(ChildAge)2:1     0.354180   0.512392   0.691   0.4894
as.factor(ChildAge)2:2     0.817766   0.449881   1.818   0.0691 .
as.factor(ChildAge)2:3     4.262111   2.380247   1.791   0.0734 .
as.factor(ChildAge)3:1     0.639622   0.482771   1.325   0.1852
as.factor(ChildAge)3:2     0.387879   0.479723   0.809   0.4188
as.factor(ChildAge)3:3     2.883054   2.027327   1.422   0.1550
as.factor(ChildAge)4:1     0.312277   0.513220   0.608   0.5429
as.factor(ChildAge)4:2     0.392627   0.486661   0.807   0.4198
as.factor(ChildAge)4:3     2.719588   2.133309   1.275   0.2024
as.factor(ChildAge)5:1     0.241178   0.529019   0.456   0.6485
as.factor(ChildAge)5:2    -0.021063   0.556103  -0.038   0.9698
as.factor(ChildAge)5:3   -10.605999 109.183758  -0.097   0.9226
---
Signif. codes:  0 ‘***’ 0.001 ‘**’ 0.01 ‘*’ 0.05 ‘.’ 0.1 ‘ ’ 1

Names of linear predictors: logitlink(mu1), logitlink(mu2), loglink(oratio)

Residual deviance: 875.3291 on 7014 degrees of freedom

Log-likelihood: NA on 7014 degrees of freedom

Number of Fisher scoring iterations: 27
```
